# Mid-Infrared Sensing of Organic Pollutants in Aqueous Environments

**DOI:** 10.3390/s90806232

**Published:** 2009-08-06

**Authors:** Bobby Pejcic, Matthew Myers, Andrew Ross

**Affiliations:** CSIRO Petroleum, P.O. Box 1130, Bentley, WA, 6102, Australia; E-Mails: matt.myers@csiro.au; andrew.ross@csiro.au

**Keywords:** mid-infrared sensor, ATR, hydrocarbon, environmental monitoring

## Abstract

The development of chemical sensors for monitoring the levels of organic pollutants in the aquatic environment has received a great deal of attention in recent decades. In particular, the mid-infrared (MIR) sensor based on attenuated total reflectance (ATR) is a promising analytical tool that has been used to detect a variety of hydrocarbon compounds (i.e., aromatics, alkyl halides, phenols, etc.) dissolved in water. It has been shown that under certain conditions the MIR-ATR sensor is capable of achieving detection limits in the 10–100 ppb concentration range. Since the infrared spectral features of every single organic molecule are unique, the sensor is highly selective, making it possible to distinguish between many different analytes simultaneously. This review paper discusses some of the parameters (i.e., membrane type, film thickness, conditioning) that dictate MIR-ATR sensor response. The performance of various chemoselective membranes which are used in the fabrication of the sensor will be evaluated. Some of the challenges associated with long-term environmental monitoring are also discussed.

## 1. Introduction

**Figure 1.**
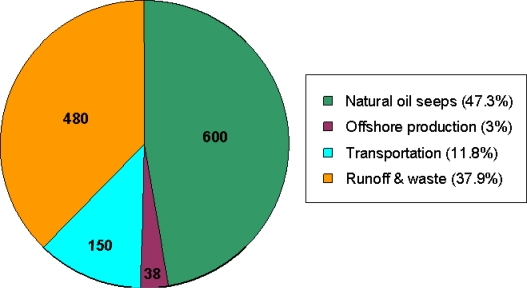
Sources of oil input into the oceans (in thousands of tons) [4].

Water is an essential commodity for all aspects of life and preserving the world’s natural water resources is one of the key issues of the 21^st^ century. In particular, there is growing concern about the impact of pollutants on the environment. Water contamination by organic substances is widespread and a great deal of effort has been devoted towards monitoring various hydrocarbons [1,2]. It is well known that hydrocarbons are a large group of compounds which have both natural and anthropogenic origins. Presently, the world is heavily reliant on hydrocarbons derived from crude oil and there is considerable interest towards increasing oil production in order to meet future world demands. However, a significant amount of hydrocarbons enter the water system through various human activities. Figure 1 shows typical levels of oil (in kilotons) released into the oceans worldwide each year from several different sources. Despite a great deal of research, information about the impact of a number of organic pollutants is unknown since a vast majority of existing and new chemicals are not routinely monitored in environmental media [3].

Addressing timely and critical water pollution issues requires sophisticated analytical tools that are both sensitive and selective. A range of chromatographic and spectroscopic technologies have been developed over the past century for monitoring a vast range of chemicals [5]. In most cases, water samples are collected from the field at various intervals by trained personnel and brought back to the laboratory where they are analyzed. Although useful information can be obtained from periodic sampling, it is widely recognized that this method is inadequate in terms of both spatial and temporal resolution. To alleviate the problem of undersampling, research has shifted towards the development of sensors that allow continuous *in situ* monitoring over long periods of time. Recent reviews of the subject have shown that chemical sensors and portable analysers play a pivotal role in the monitoring of oceans, lakes, and rivers [6,7]. In particular, sensors for dissolved oxygen, conductivity, and pH are routinely deployed to monitor and understand the aquatic environment.

Chemical sensors for the detection of organic substances in aquatic environments are less frequently used. Although, a range of sensors have been developed for hydrocarbons [8], very few appear to be suitable for the determination of dissolved hydrocarbons in marine water. One of the major challenges when detecting hydrocarbons in natural waters is the presence of ionic salts and organic matter (i.e., humic acids, etc) which can interfere with the sensor response. A number of sensors (i.e., piezoelectric, electrochemical) respond to changes in salinity and ionic content, and the levels can vary significantly from one region to another. Others (i.e., fluorescence) are affected by the presence of humic substances and their removal or separation is usually necessary prior to analysis. A sensor that can directly analyse hydrocarbons in a wide range of aquatic environments (i.e., seawater, lake, etc) with minimal sample perturbation would be invaluable.

One particular analytical tool that does not appear to be severely affected by chemical interferences and turbidity is the mid-infrared (MIR) sensor based on attenuated total reflectance (ATR). Historically, infrared spectroscopy (IR) has been used to provide structural and compositional information on a wide range of inorganic and organic molecules. In recent years there has been a shift towards the use of IR for the detection of a number of environmentally relevant pollutants in water and this has paved the way for a wide range of applications in biomedical [9] and process monitoring [10]. Since a number of articles have been published in this area, it seems timely to review the development of IR-based sensors in addressing the issues and challenges facing environmental monitoring of hydrocarbon contaminants. The most significant advances in relation to MIR-ATR sensing of organic compounds in aqueous environments will be reviewed. The article will pay particular attention to sensor design and type of materials used to prepare the sensing surface.

## 2. Principles of Attenuated Total Reflectance (ATR)

Since the development of the Fourier transform infrared (FTIR) spectrometer in the early 1960s there has been a significant rise in the application of infrared spectroscopy to investigate and understand a wide range of problems [11]. The majority of the infrared studies performed in the past involved collecting IR spectra in the direct transmission mode. However, in recent years a number of accessories and refection-based methods have been developed, and these have extended the capability of FTIR to measure a wide range of complex samples in the laboratory. In particular, attenuated total reflectance infrared spectroscopy is one of only a few techniques that allows the interfacial phenomena of many important chemical systems and materials to be investigated *in situ* [12]. Although, ATR has been around for several decades, the technique has only recently been exploited as a tool for chemical sensing.

ATR, also known as internal reflection spectroscopy (IRS) or evanescent field spectroscopy (EFS), is a versatile and non-destructive technique. To reduce confusion, the term ATR will be used exclusively throughout this entire manuscript. When light strikes an interface between two materials of different refractive indices some of the light will be reflected and some will be transmitted. A standing wave normal to the reflecting surface is established in the denser medium and an evanescent non-propagating field in the rarer medium. An optically transparent material of high refractive index, known as the internal reflection element (IRE), is used to ensure that the IR beam propagates through a series of internal reflections at the sample/IRE interface. Although complete internal reflection occurs at the interface, some of the radiation (i.e., evanescent wave) does penetrate into the sample, noting that the sample is in direct contact with the IRE. Only molecules in the region of the evanescent wave will undergo interaction with the IR radiation, since the evanescent wave decays exponentially in amplitude with distance from the IRE surface into the adjacent sample. The depth of penetration (*d_p_*) of the IR radiation into the sample can be mathematically described by the following equation:

(1) dp=λ2πn1[sin2 θ−(n2n1)2]12

where *λ* is the wavelength of light, *θ* is the angle of incidence, and *n_1_* and *n_2_* are the refractive indices of the IRE and sample, respectively [13]. The penetration depth is proportional to the wavelength of the infrared beam and it is greater for a sample with a higher refractive index.

A wide variety of materials are commercially available for use as IREs. The paper by Vigano *et al.* [14] compares and contrasts various crystalline inorganic-based materials that have been used for generating an evanescent wave. Despite the availability of several crystals, zinc selenide (ZnSe) is the most commonly used since it is relatively inexpensive and transparent over a wide IR range. The IRE, or sometimes known as the waveguide, can be either in the form of a trapezoidal crystal (i.e., ZnSe) that is typically 80 mm long and 4 mm thick or an optical fiber (i.e., silver halides, chalcogenides) that has a length of several meters. Although, very little is reported on the most optimum waveguide length for MIR sensing of hydrocarbons, most use an IRE that has between 6 to 8 reflections at the sample/IRE interface. The total number of reflections can be modified by varying the geometry of the IRE and the angle of incidence. If the IRE length and thickness is *l_IRE_* and *t*, respectively, then the total number of reflections (*N*) can be determined by the following equation [13]:

(2) $$N=\left(\frac{l_{\mathrm{IRE}}}{t}\right)\text{cot}\;\theta$$

In principle, a longer IRE should lead to a significant improvement in sensor sensitivity since the sampling area along the waveguide and the total sample pathlength is greater due to more internal reflections. According to the Beer-Lambert law the absorbance (*A*) is directly related to the sample pathlength (*l*):

(3) A=εcl

where *c* is the analyte concentration and *ε* is molar extinction coefficient. The above equation implies that when the pathlength of the IR light through a material increases then the interaction between the IR radiation and the analyte molecules which are present in the sample will also increase. Recent studies by Roy and Mielczarski [15] have shown that by increasing the number of internal reflections of a ZnSe element from 8 to 50 can lead to nearly a six-fold improvement in the detection limit.

An optical fiber is capable of achieving many more internal reflections and it has been suggested that this could give rise to lower detection limits [16]. Table 1 compares the detection limits obtained on various IR sensors for toluene and it seems that the fiber optic is less sensitive relative to the trapezoidal ZnSe IRE. This may not be surprising since the sensitivity is governed by factors other than just the geometry of the IRE and these will be discussed in the proceeding sections. However, it is important to note that attenuation losses become an issue with increasing waveguide length and Gobel *et al.* [17] propose the use of tapered fibers rather than a longer fiber as a method of lowering the detection limit of the MIR sensor. There is no doubt that more internal reflections enhances the probability that the evanescent wave will interact with the target analyte, however, there will be a trade-off since the distribution/dispersion of the analyte molecule at a given concentration is much less over a larger sampling area and/or volume. Nevertheless, this article will focus mainly on the MIR-ATR sensor that employs a trapezoidal IRE, noting that various reviews have been recently published discussing the development and application of optical fibers for mid-IR sensing [18,19].

### 2.1. Sensor Design Criteria

**Figure 2.**
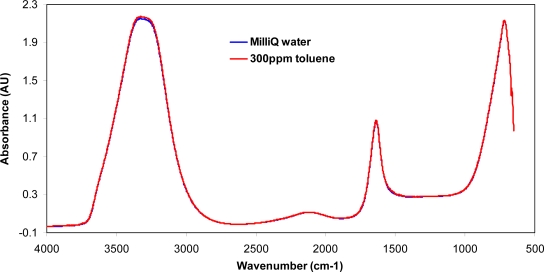
Comparison of the mid-infrared spectra of a ZnSe IRE exposed to MilliQ water in the absence and presence of 300 ppm toluene.

One of the major challenges when passing IR radiation through an aqueous solution is the strong absorbance and interference of water. It is evident that the mid-infrared spectrums of a ZnSe IRE exposed to an aqueous solution with and without 300 ppm toluene are almost identical (see Figure 2), suggesting that the FTIR is unable to detect toluene at such levels when the IRE is immersed directly in water.

This presents a significant challenge for the analyst since most hydrocarbons in natural waters (i.e., rivers, lakes) are typically present in the parts per billion to the parts per million range. Normally, when measuring organic compounds at low concentrations (i.e., less than ppm) the sample is usually exposed to some membrane that selectively extracts the analyte molecule followed by a removal or desorption step prior to detection [24].

**Figure 3.**
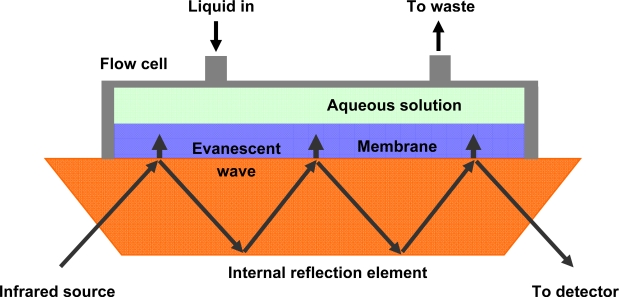
Hydrocarbon detection using an MIR-ATR sensor.

It has been shown recently that the detection of low levels of hydrocarbons in aqueous solutions by ATR-FTIR is viable provided that the surface of the IRE is chemically modified with an appropriate material, noting that this concept is based on solid-phase microextraction [25]. A membrane that is less than 50 μm thick is placed directly onto the IRE and Figure 3 shows a typical sensor configuration / experimental setup. A thin layer is used to ensure rapid and reversible response and by tailoring the chemical properties of the membrane it can be made to preferentially interact with certain hydrocarbon compounds. Usually, the coated IRE is placed in a flow cell which comprises a peristaltic or piston pump to deliver the aqueous solution to the sensing surface. Analyte molecules are extracted into the film and equilibration is achieved more rapidly when the aqueous solution is flowed across the surface. In most environmental studies the components are constructed of stainless steel in order to minimize analyte adsorption onto the walls of the flow cell. Detection is commonly achieved with a liquid-nitrogen cooled mercury-cadmium-telluride (MCT) photoconductor, which monitors the infrared radiation that passes through the IRE. Other detectors are available in the MIR range (i.e., triglycine sulfate pyroelectric detector, etc), however, the MCT detector is generally preferred since it is more sensitive [11].

The design of an IR sensor for the detection of organic compounds in aqueous environments requires the use of hydrophobic coatings and membrane materials. Despite a number of different types of materials being available for binding with hydrocarbons [8], polymers appear to be the most promising in terms of achieving acceptable sensitivity and reproducibility. The main objectives of the membrane are to (1) exclude the water/interfering molecules from the analytical volume that is probed by the evanescent field and (2) to reversibly and selectively extract the desired target hydrocarbon molecule. Although, the chemical (i.e., forces of interaction) and physical properties (i.e., porosity) of the coating play an important role on the analytical performance of the sensor (i.e., selectivity, sensitivity and response time), the chemoselective material used must not have IR absorption bands in the region which are characteristic of the analyte molecule. Furthermore, a membrane with a lower refractive index relative to the IRE is recommended to ensure that a significant portion of the IR beam reaches the MCT detector. Finally, the membrane needs to be insoluble in water and in intimate contact with the IRE so that an analytically useful signal can be generated.

Ensuring intimate contact between the IRE and the membrane is one of the main challenges encountered when designing the MIR-ATR sensor. In most cases the IRE surface is prepared by dissolving the material to be coated in an organic solvent and using the drop cast method to obtain a thin and uniform film. Normally a volatile solvent is used to ensure complete evaporation prior to analysis. The solvent is usually allowed to evaporate overnight at room temperature, however, some studies employ a heat curing method to remove the solvent and to help improve the film-IRE adhesion properties. Although, very little is reported on which is the most suitable method for preparing the sensing surface, various research papers have recently appeared showing that the conditions (i.e., concentration, solvent, etc) used during film formation can play a significant role on the coating quality and surface morphology [26,27].

### 2.2. Factors Influencing Sensor Response

Membranes, which allow elevated rates of diffusion and enhanced levels of analyte absorption, are useful from a sensing point of view. The type of material used to coat the IRE has a significant influence on the analytical performance of the sensor. A great deal of research has been undertaken in developing polymeric membranes for ATR sensing, and Table 2 compares various polymers and conditions used to prepare the IRE surface. It is evident that the sensitivity depends on the nature of the polymer and the film thickness. One of the advantages of using polymers is that thin uniform films can be readily attained, noting that the film thickness is controlled by changing the polymer solution concentration.

A number of groups have studied the MIR sensor response as a function of polymer film thickness. Gobel *et al.* [28] investigated the influence of polyisobutylene (PIB) thickness in the range 10.1–37.7 μm and found that the absorbance signal arising from chlorobenzene absorption was highest at 10 μm. By contrast, Roy and Mielczarski [15] showed that the greatest signal intensity of various chlorinated hydrocarbons absorbed into the polymer poly(ethyl-co-propylene) was achieved using a 1 μm film. Others suggest that a film thickness of ∼4 μm can provide detection limits in the ppb range [20]. Generally, films that are between 1 to 10 μm thick give the most optimum analytical performance, however, this will depend on the region of the IR spectrum used since the depth of penetration and the wavelength of the infrared beam varies according to Equation 1. It is worth noting that the film thickness does not only play an important role on the sensor sensitivity, but also on its response time. If the MIR-ATR sensor is to be used for discrete or continuous monitoring, then the manner in which it is deployed will depend on the time taken to reach equilibrium. Typically, the response time increases with film thickness and the absorbance signal is dictated by diffusion of the hydrocarbon molecule through the polymer network. Despite significant variations in the literature on the most appropriate film thickness it is recommended that the coating be roughly three times the penetration depth in order to ensure optimum sensor performance.

Various forces (i.e., Van der Waals, dipole-dipole) play an important role in the interaction between the hydrocarbon molecule and the polymer. The hydrophobic nature of some polymers ensures that mainly non-polar compounds are absorbed into the film. Grate and Abraham [31] established that most hydrocarbons undergo solubility interactions with polymeric materials and developed a linear solvation energy relationship to quantify the interactions. The amount of analyte dissolved in the polymer film depends on the distribution of the substance between water and the polymer. The partition coefficient (*K*) is often used to quantitatively describe the process and is defined as the ratio of the equilibrium concentration of the analyte in the coating, [*A*]*_film_*, to its equilibrium concentration in solution, [*A*]*_aq_*, and is given as follows:

(4) $$K=\frac{{\left[A\right]}_{\mathrm{film}}}{{\left[A\right]}_{\mathrm{aq}}}$$

Generally, membranes that have a *K* value of greater than 100 are useful from a hydrocarbon sensing point of view [32]. The type of phase (i.e., gas versus liquid) in contact with the membrane also plays a significant role on the partitioning process, noting that the partition coefficients for many non-polar hydrocarbons are generally higher in the gas phase compared to aqueous solutions [33]. A number of groups have determined the partition coefficient of several different organic compounds in various polymers and the accuracy seems to depend significantly on the chosen analytical method and the type of sorbate to be detected [32,34]. Others suggest that the experimentally determined partition coefficient can vary as function of the membrane film thickness [25]. Langenfeld and coworkers showed that the partition coefficient of various hydrocarbons into polydimethylsiloxane was generally higher for a 7 μm thick film relative to a 100 μm coating [25]. This is surprising considering that the partition coefficient is governed solely by the thermodynamics of a particular system and is expected to be independent of film thickness. The variation in K with film thickness implies that equilibrium was not reached, however, no differences were observed in the measured partition coefficient after a 5 hour equilibration and one that was equilibrated for 1 week. It was concluded that there may be some subtle differences in the physical or chemical properties of the various films due to variations in the sorbent preparation method.

The partition coefficient is not the only parameter that determines the success of hydrocarbon extraction into a polymeric material. Gobel *et al*. [28] found that the most suitable polymer for extraction of hydrocarbons was not necessarily the one that had the highest partition coefficient. They revealed that the relationship between the hydrocarbon-polymer partition coefficient and the MIR sensor sensitivity was not proportional. Others also observed no linear relationship and concluded that the partition coefficient is not sufficient enough to completely describe the analyte-polymer system [29]. The diffusion of hydrocarbon molecules into and through the polymer network was also found to be a crucial factor when evaluating the detection limit of the MIR-ATR sensor. A number of studies determined the diffusion coefficients of several hydrocarbons in various polymers and showed that this is another important parameter to consider when selecting a particular membrane [29,35–38]. McLoughlin and coworkers described the diffusion profile of various chlorinated and aromatic hydrocarbons through a Teflon film, and showed that they obey Fick’s second law of diffusion [39]. The rate of diffusion of a hydrocarbon molecule through a polymer membrane will depend on its size and structure along with the physicochemical properties of the polymer (i.e., pore size, etc). Generally, the diffusion coefficient varies inversely with the molecular size and the smaller the molecule the faster it diffuses. However, other factors such as hydrogen bonding interactions between a molecule along with intermolecular interactions between the polymer and the penetrant are also expected to play a significant role on the diffusion process. Flavin *et al.* [29] measured the diffusion properties of various hydrocarbons and showed that some polar molecules do not follow the general trend between molecular cross-section and diffusion coefficient, implying that intermolecular hydrogen bonding gives rise to molecular clustering and this is responsible for an increase in the molecular size and a lower diffusion coefficient than expected. Although, Fick’s second law has been applied to a number of different solvent-polymer systems, many studies fail to take into account swelling and convection processes [40].

The partition and diffusion coefficients are useful quantitative parameters when describing the interaction between a particular hydrocarbon molecule and a polymer membrane. A great deal of research has been undertaken to understand the influence of these on the response characteristics (i.e., limit of detection, response time, etc) of a polymer coated MIR-ATR sensor. Polymers that have a low density, low glass transition temperature (*T_g_*), and an amorphous structure appear to be more successful for the extraction of hydrocarbon compounds [41,42]. Generally, when the *T_g_* is low the free volume is higher between the chains and this is responsible for the much greater hydrocarbon diffusion/transport and sorption. The free volume size and distribution in the polymer depends on various factors such as the polymer molecular weight, structure/type of atoms, crystallinity, solvent used during film preparation, etc. Crystalline polymers are usually more compact making it much more difficult for substances to diffuse through. Yang and Huang [43] studied the diffusion and absorption properties of water and several aromatic-based compounds in various polystyrene derivatives. They revealed that the water and hydrocarbon permeability increased with an increasing alkyl chain length on polystyrene and explained this in terms of a lower crystallinity, noting that the *T_g_* of alkylated polystyrene decreases with increasing chain length. Others compared the diffusion coefficient of toluene in various polymers and concluded that the polymer pore size plays a major role on molecular diffusion rather than the *T_g_* value [29]. Despite a significant amount of research there still appears to be a lack of fundamental understanding on the effect of the polymer structure (i.e., pore space, morphology, etc) on the sensor response mechanism. The free volume size and distribution in the polymer has a profound influence on the sensor sensitivity for hydrocarbons, however, most of the reports have failed to probe or examine the free volume properties.

## 3. Environmental Water Monitoring

Assessing the impact of hydrocarbon pollutants on the aquatic environment is not a simple task considering the many different types of hydrocarbons and the number of different pathways for a pollutant to enter a water body (i.e., river, ground, etc). Hydrocarbons (i.e., aromatics, alkanes, alkyl halides, etc) display a wide range of solubilities and these depend on a number of environmental conditions (i.e., temperature, salinity, natural organic matter, etc) [44]. In addition, various factors (i.e., hydrolysis, photolysis, oxidation, biodegradation) play a significant role on the distribution and transformation of many organic substances. Long-term observations of the aquatic environment are essential in understanding any subtle changes and distinguishing between natural and anthropogenic processes. A sensor system that can provide continuous data on the water quality and can be operated for extended periods with minimal need for re-calibration would be extremely useful. The MIR-ATR sensor is a promising analytical tool [45], however, it is unclear which chemoselective material is the most suitable for the detection of hydrocarbon compounds in water. One of the objectives of this manuscript is to evaluate the analytical performance (i.e., sensitivity, response time, stability, etc) of various materials which have been used to sense hydrocarbons. Since many organic substances have been detected using the MIR-ATR sensor the review will focus on a few groups which are responsible for a majority of the hydrocarbon pollutants that enter the water system.

### 3.1. Aromatic Hydrocarbons

Aromatic hydrocarbons belong to a large group of organic compounds which display a wide range of properties. Traditionally, they are divided into the following two classes: monocyclic and polycyclic. The monocyclic group comprise a single ring of six carbon atoms that are connected by delocalised electrons into a regular hexagonal structure. Benzene, toluene, ethylbenzene, and xylene are common monocyclic aromatic hydrocarbons and are sometimes referred to as BTEX. This group of compounds are well known to cause serious health concerns and need to be regularly monitored. For instance, benzene is carcinogenic to humans and acute exposure to high levels is known to affect the central nervous system. Similarly, prolonged exposure to toluene can have adverse health effects. The recommended safe levels vary significantly from one country to the next, however, the World Health Organization (WHO) suggests that benzene levels in drinking water should not exceed 0.01 ppm, whereas the WHO guideline value for toluene is 0.7 ppm [46]. Ethylbenzene and xylene are generally considered to be less toxic to humans than benzene, and the recommended ethylbenzene and xylene levels in drinking water are <0.3 ppm and <0.5 ppm, respectively [46]. The relatively higher water solubility of BTEX compounds compared to saturated hydrocarbons (i.e., methane, propane, hexane, etc) means that they display a greater tendency to spread in contaminated water [47]. Industrial waste, leakage, and spills are typical sources of water contamination, however, a significant portion of BTEX compounds enter the water system from atmospheric pollution.

**Figure 4.**
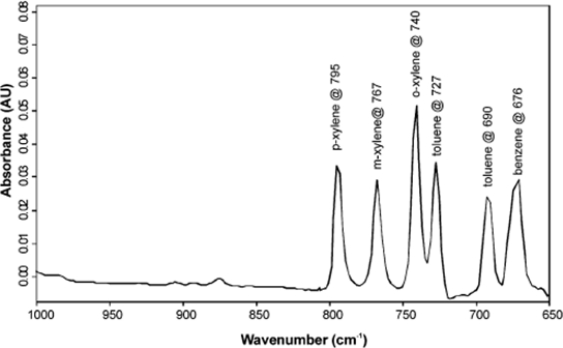
The simultaneous determination of several aromatic compounds by MIR-ATR sensor. Reproduced from [20] with the permission of the American Chemical Society.

Although, a number of sensors have been developed over the past several decades to directly measure BTEX levels in water, many of them are unsuitable for environmental monitoring since they struggle to clearly resolve and separate the signals arising from the various aromatic components (i.e., benzene, toluene, ethylbenzene and xylene). Their similar structure and chemical properties presents a significant challenge for signal discrimination which cannot be easily accomplished by employing a selective membrane or pattern recognition/multicomponent analysis methods. However, the MIR-ATR sensor is able to easily overcome the problem of limited selectivity by resolving the total hydrocarbon signal according to their individual characteristic infrared absorption frequencies. Recently, Mizaikoff and coworkers developed a MIR-ATR sensor based on a ethylene-propylene copolymer and managed to simultaneously distinguish between 5 compounds (i.e., benzene, toluene, p-xylene, o-xylene, and m-xylene) in less than 20 min [20]. Figure 4 displays the IR spectrum of the compounds at 500 ppb levels, noting that the peaks are well resolved and separated. The ability to perform multianalyte detection using characteristic IR frequencies is an attractive feature of the MIR-ATR sensor.

Water diffusion and swelling often gives rise to a broad absorption band and baseline drift in the region of interest. To ensure a stable baseline, the ethylene-propylene coated IRE was immersed in deionised water for a period of 1 day prior to analysis. When reliable experimental protocols are employed benzene, toluene and xylene can be easily detected down to 45, 80, and 20 ppb levels, respectively. Furthermore, the MIR-ATR sensor was shown to respond linearly to the various aromatic hydrocarbons over a wide concentration range (i.e., 0–1,000 ppb). Although, the performance of the MIR-ATR sensor is comparable to other standard methods for BTEX analysis, the long term stability of the sensor was not evaluated and further work is needed to determine if soaking the sensor in water for extended periods (>1 day) influences its sensitivity.

Long term continuous monitoring requires membranes that are robust and chemically stable in environmental waters. Polyvinyl chloride (PVC) is a common thermoplastic material which does not undergo significant degradation and oxidation after exposure to water. It comes as no surprise that a number of groups have investigated PVC as a possible receptor material for the detection of hydrocarbons in water. One of the major challenges when developing PVC films that are of micron thickness is the slow diffusion of analyte molecules to the sensing surface, noting that PVC is a rigid polymer with a high glass transition temperature. To improve the rate of analyte diffusion and transport, Regan and coworkers investigated a range of plasticised PVC polymer membranes and found that the plasticiser type and concentration have a profound influence on the sensor response to BTEX compounds [48]. It was shown that hydrocarbon absorption and enrichment is much greater in the presence of a plasticiser. Equilibrium was reported to be reached in less than 8 min for the individually measured analytes, however, no sensor measurements were made comparing the response times in the presence and absence of the plasticiser. It is well established that the introduction of a plasticiser can create spaces and free volumes in the polymer network and this helps improve the rate of analyte diffusion. Silva *et al.* [22] also observed significant enhancement in BTEX sensitivity when doping the PVC membrane with the plasticiser di-2-ethylhexylphthalate. Detection limits of 5, 6.9, 9 and 4 ppm were achieved for benzene, toluene, ethylbenzene and xylene, respectively, which represents a substantial improvement in sensitivity considering that the transmittance method was employed during measurement. The ability to modify the properties of PVC films is an attractive feature of this material; however, further studies are needed to investigate sensor film robustness and plasticiser leaching effects. The high detection limits obtained on a PVC coating suggests that this membrane may not be particularly sensitive for monitoring of BTEX compounds in drinking water.

Obviously polymeric membranes that can measure BTEX compounds in water down to ppb levels would be extremely useful. Polyisobutylene (PIB) is another promising material that has very little or no IR bands in the 900–650 cm^−1^ region, which is the relevant part of the spectrum for the analytical detection of BTEX. Yang and Tsai evaluated the response of polyisobutylene in an aqueous solution comprising toluene and obtained a detection limit of 337 ppb [30]. Rather than expose the sensor surface directly to the aqueous solution, they sampled the headspace. This was achieved by heating the solution to liberate the BTEX compounds, followed by a cooling step prior to MIR detection. This method has the advantage of reducing problems relating to membrane instability / degradation during long term exposure to aqueous solutions. It would appear that this approach works only for volatile organic compounds, however, it was demonstrated that other less volatile hydrocarbons (i.e., chlorobenzene) can also be easily detected in the ppb range. By contrast, Flavin *et al.* [16] developed a Teflon coated MIR sensor and report a limit of detection for toluene and ethylbenzene of 27 and 24 ppb, respectively. One of the main advantages of using a Teflon membrane is that it does not undergo significant degradation and changes in the hydrocarbon permeation properties after long term use. The sensor surface was prepared by spin coating Teflon onto the IRE followed by a heat curing protocol to improve the film properties. The high sensitivity is remarkable considering that only six reflections were employed at the polymer/IRE interface.

Polycyclic aromatic hydrocarbons (PAHs) are a group of ubiquitous contaminants which also have adverse effects on humans and marine species at very low concentrations [44,49]. These are a complex class of condensed multi-ring benzenoid compounds and according to some reports biomass is a large contributor of PAH emissions, followed by coal combustion, and coke production [50]. Fluorescence-based sensors are generally the preferred method for the detection of PAHs in the environment due to their much greater sensitivity [51]. Although, these sensors are extremely sensitive, they cannot easily separate the analytical signal arising from the target molecule in the presence of other aromatic compounds, noting that the aquatic environment also contains phenols, humic acids, etc at significant levels. Interestingly, the development of a MIR-ATR sensor has also been explored as an alternative analytical tool for monitoring PAHs. Yang and Huang [43] compared the performance of various polymers for the detection of chloronaphthalene in aqueous solution. They showed that a long alkyl chain (C12) *para*-substituted polystyrene leads to a material with vastly improved sensitivity for PAHs. The improved response was rationalized in terms of lower polymer crystallinity rather than a more hydrophobic polymer as a result of the attached alkyl group. However, it was revealed that the diffusion of chloronaphthalene into the alkylated polystyrene is very slow (∼1 h), despite a film thickness of 4 μm. The sluggish response may not be surprising considering that chloronaphthalene is a large molecule compared to BTEX compounds, noting that the diffusion coefficient is strongly influenced by the molecular size. It is evident that mid-IR sensing of PAHs is possible, however, it appears that polystyrene is not sensitive enough and further studies are needed to evaluate the performance of other membranes for the detection of PAH compounds.

### 3.2. Halogenated Hydrocarbons

Hydrocarbons that contain a halogen atom (i.e., F, Cl, etc) also pose a severe threat to the environment and are known to cause serious health effects in humans after prolonged exposure [44,46]. This group of hydrocarbons are used by industry as a primary source for the manufacture of a wide range of chemicals and generally enter water bodies through spills and atmospheric pollution. In addition, some halogenated hydrocarbons are generated at significant levels through chlorination, which is a major treatment process used in many domestic water supplies and swimming pools. The reaction between chlorine and natural organic matter form a range of unwanted products (i.e., trihalomethanes, etc) that are responsible for various health issues [52]. Subsequently, the regulation of chlorinated hydrocarbon levels in drinking water is an area that has received a great deal of attention over the past several decades. Table 3 summarizes some of the halogenated hydrocarbons that are considered by the WHO to be toxic and need to be regularly monitored. Although, gas chromatography-mass spectrometry (GC-MS) and high performance liquid chromatography (HPLC) are conventionally used for determining the concentration of these and other halogenated hydrocarbons in water, the MIR sensor has become an attractive tool that can provide direct quantitative information in a timely manner.

Halogenated hydrocarbons comprise a large group of highly water soluble compounds (see Table 3), however, most of the interest has been directed to only a small select few. In particular, tetrachloroethylene (C_2_Cl_4_) is a volatile solvent commonly used in dry cleaning operations and industrial metal cleaning / finishing. At high concentrations C_2_Cl_4_ is responsible for central nervous system depression along with kidney and liver damage, and it is recommended that the tetrachloroethylene levels in drinking water should not exceed 40 ppb [46]. Several groups have investigated the performance of a number of polymers for the determination of tetrachloroethylene in water. Polyethylene is one particular material that is ideal for sensing chlorinated hydrocarbons since it has very few absorption bands in the mid-IR region. Heinrich *et al.* [42] evaluated the enrichment properties of various polyethylene polymers and observed a relationship between polymer density and sensitivity. A detection limit of 0.2 ppm and 2.5 ppm is reported for the low (i.e., 0.89 g/cm^3^) and high (i.e., 0.96 g/cm^3^) density polyethylene, respectively [42]. Although, the low density polyethylene is more sensitive for C_2_Cl_4_, further work is still needed to improve its slow response time [42]. Likewise, Gobel and coworkers evaluated the response of low density polyethylene and showed that it was less sensitive for C_2_Cl_4_ compared to PIB despite a much lower *T_g_* [28]. Unfortunately, no detection limits are reported, noting that the absorbance signal arising from the extraction of C_2_Cl_4_ into various polymers with time was measured and compared. The authors concluded that PIB is better suited for the detection of tetrachloroethylene in a mixture comprising various chlorinated hydrocarbons, despite showing that the C_2_Cl_4_ diffusion and partition properties being more favourable in a ethylene-propylene copolymer [28,37]. Indeed, other workers achieved a detection limit of 19.6 ppb with ethylene-propylene copolymer [15], implying that this membrane may be more suitable for monitoring tetrachloroethylene in drinking water.

Chloroform (CHCl_3_) is another compound which has received a great deal of attention partly because of its carcinogenic properties. It has been suggested that levels of greater than 300 ppb in drinking water are considered to be unsafe for human consumption [46]. Monitoring the concentration of chloroform in water by MIR-ATR can be difficult due to its relatively high water solubility [47] and its generally low partition coefficient [28]. Early work by various groups established the experimental conditions necessary for the detection of chloroform and as a result obtained detection limits in the low ppm range. Heinrich *et al.* [42] report a detection limit of 10.5 ppm on an IRE coated with trichloroctadecylsilane, whereas Gobel *et al.* [28] used PIB and achieved a limit of detection of 1.5 ppm. Although, linear calibrations were obtained in the concentration range of 5 to 100 ppm with PIB [28], it is evident that this polymer is not particularly sensitive under these conditions. By contrast, Roy and Mielczarski [15] demonstrated that a MIR sensor comprising a film of poly(ethyl-co-propylene) may be more suitable for detecting ppb levels of chloroform in water. A detection limit of 306 ppb was obtained for a 1 μm thick coating with an IRE that employs eight reflections. Interestingly, the authors managed to improve the sensitivity even further by increasing the number of reflections to 50 and report a detection limit of ∼49 ppb [15]. Other workers investigated the diffusion of chloroform and showed that the diffusion coefficient was almost 10 times higher in the ethylene-propylene copolymer compared to PIB [38]. Although, there are many polymers available for chloroform extraction, it appears that poly(ethyl-co-propylene) is the most sensitive and is able to achieve ppb detection limits.

It is evident that a significant majority of the research on the MIR sensor has focussed on pushing the sensitivity down to ppb detection levels. Although, sensitivity is an important consideration when selecting a sensor, it is not the only property that determines its success. Others have shown that the MIR-ATR sensor is useful for distinguishing between many different chlorinated hydrocarbons simultaneously. In particular, Kraft and Mizaikoff [53] demonstrated that a ethylene-propylene copolymer can be used to monitor a wide range of chlorinated hydrocarbons (i.e., dichlorobenzene, chlorobenzene, trichloroethylene, tetrachloroethylene, dichloroethylene, chloroform) in saline water in less than 20 mins. The signals arising from the six different chlorinated hydrocarbons were separated and detected down to ppb levels. By carefully selecting sufficiently separated absorption bands, linear calibration curves were obtained over a wide concentration range (i.e., ∼0.1 to 5 ppm). Studies were also carried out to investigate the influence of salinity, turbidity and humic acid, and these did not present any significant problems. Similarly, aliphatic and aromatic hydrocarbon interferences were negligible suggesting that the sensor can be reliably used to analyse chlorinated hydrocarbons in complex environmental samples.

### 3.3. Other Organic Compounds

Phenols are another large group of organic compounds that are present at significant levels in the environment and a number of them frequently come under the scrutiny of environmental regulation authorities. According to the WHO a number of chlorophenols are considered to be toxic and pose a health threat [46]. For instance, 2,4,6-trichlorophenol and pentachlorophenol are reported to be carcinogenic and a concentration value in drinking water of greater than 0.2 ppm and 0.009 ppm, respectively is deemed to be hazardous [46]. A sensor that can rapidly and accurately measure these and many other phenol compounds will be an extremely useful surveillance tool. Yang and coworkers evaluated the response of various polymeric materials for the detection of several types of phenols with different attached groups (i.e., hydroxyl, nitro, methyl, chlorine) [54, 55]. A polyacrylonitrile-co-butadiene (PAB) coating was found to be the most sensitive with detection limits ranging between 100 to 600 ppb for a majority of the phenols. The performance of PAB is not surprising considering that the presence of a polar cyano group is partly responsible for interacting with the -OH group in phenol. However, the detection limits for phenol, 3-hydroxylphenol and 2,4-dinitrophenol were much higher (i.e., >1 ppm), suggesting that this membrane may not be suitable for the determination of slightly more polar compounds. As expected the pH and the solution ionic strength were shown to have a significant effect on the phenol-polymer partitioning process. Interestingly, it was found that the interaction between 2,3-dichlorophenol and a polyacrylonitrile coating did not generate a strong IR signal implying that π-π interactions may also play a role in the extraction of phenols [55]. To demonstrate the application of a PAB coated MIR-ATR sensor the authors analyzed real water samples collected from a creek and an underground water supply located in the northern part of Taiwan. Despite displaying some promising results for the detection of phenolic compounds, further studies are needed to evaluate its reliability against standard analytical methods.

Hydrophobic polymers have been successfully used to sense a wide range of non-polar hydrocarbon compounds in water. However, the detection of hydrocarbon contaminants that are slightly more polar usually requires membranes with some functionality. Various groups have developed organic-inorganic hybrid materials [56] and cyclodextrins [57] for the detection of several different polar compounds. Recently, Flavin and coworkers demonstrated that a organically modified sol-gel based on a silicon alkoxide can be used to detect p-nitrochlorobenzene down to 0.7 ppm [56]. By contrast, Yang *et al.* [57] showed that cyclodextrin interacts favourably with aromatic compounds that contain a carboxylic acid (i.e., benzoic acid) and report a detection limit of ∼100 ppb. Cyclodextrin is a promising material for detecting benzoic acid, however, further work is needed to improve sensor reversibility and response time. Although, it was shown that the sensing surface can be regenerated by changing the solution pH, noting that at pH 3 the removal of absorbed benzoic acid occurred most rapidly. It is evident that MIR sensing of polar hydrocarbon compounds (i.e., phenols, aromatic acids, nitroarenes, etc) is possible provided that the chemoselective material possesses certain functional groups (i.e., hydroxyl, etc).

## 4. Future Work

**Figure 5.**
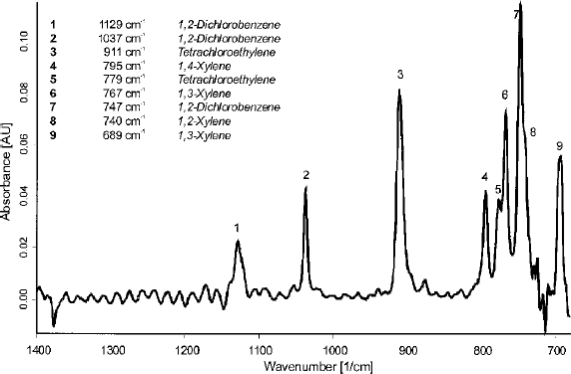
MIR-ATR sensing of various hydrocarbons from different families. Reproduced from [59] with the permission of the Society for Applied Spectroscopy.

The vast majority of the research in the field of MIR sensing has primarily focussed on the design and application of chemoselective materials. Notwithstanding, some improvements in the components required for MIR sensor systems have also been made over the past several decades and is reported elsewhere [58], The ability to distinguish between many different organic pollutants simultaneously (i.e., aromatic, phenols, alkyl halides, etc) makes the MIR sensor a unique and powerful tool for environmental monitoring [20,53,59]. Figure 5 clearly shows that several compounds from two different families can be separated and detected. Furthermore, various interferences (i.e., salinity, humic acids, turbidity, etc) do not pose a significant problem [53,59], suggesting that the sensor behaves reliably during hydrocarbon assaying of real water samples. Despite being capable of detecting many hydrocarbons over a wide concentration range (i.e., 10 ppb 500 ppm), one important issue that has not been thoroughly addressed is sensor reversibility. Many of the polymeric membranes discussed in this review seem to have response times ranging between 5–20 mins; however, most reports fail to provide data on sensor reversibility. Indeed, surface regeneration and sensor reversibility still remains a significant challenge and we propose that further studies should consider modifying the micro/nano-structure of the polymer membrane by varying the conditions used during film formation [26,27]. Similarly, very few field studies have been performed in relation to long term continuous monitoring of environmental waters (>1 day). Invariably, most sensing films gradually absorb water and some of its constituents with time, and this can lead to various chemical processes. Membrane degradation through either swelling or chemical modification is an issue that can reduce the data quality and ultimately inhibit the lifetime of the sensor. Unfortunately, many of the reports do not provide information on the MIR sensor stability over time and further studies are required to determine if the sensitivity varies when the membrane is exposed to natural waters for extended periods (>1 day). Evaluating the long term stability of the membrane is an important issue that needs to be addressed if the sensor is to be widely accepted as a standard tool for environmental monitoring.

If the MIR sensor is to be routinely deployed for long term hydrocarbon water monitoring then maintaining the integrity and quality of the membrane film is paramount. Biofouling is one of the major problems that is responsible for impacting on sensor performance and can limit the effective deployment period of a chemical sensor. This is a natural process that occurs on the surface of nearly all materials which come into contact with the marine surroundings. Microorganisms attach themselves at the solid-liquid interface and the formation of biofilms usually occurs immediately upon immersion of the sensor in the aquatic environment. In the case of membrane based chemical sensors, the attachment and growth of microorganisms on the chemoselective surface is responsible for inhibiting analyte diffusion and binding. A great deal of research is aimed at understanding the mechanism of marine biofouling and various groups have shown that biofouling can be suppressed considerably by carefully designing the membrane surface. Smith *et al.* [60] investigated the physical properties of several gas sensor membranes and demonstrated that the membrane pore size and surface roughness have a significant influence on the attachment of bacteria. However, it was revealed that these membranes provide little protection against biofouling over extended periods of exposure. Others have suggested that the surface structure and micro-texture of the material play an important role. Recent studies by Scardino *et al.* [61] have shown that the micro-textured structure of polycarbonate is responsible for impeding the attachment of certain microorganisms and by carefully tailoring the surface it can be used to alleviate biofouling problems. By contrast, the review by Genzer and Efimenko [62] highlights the importance of superhydrophobic surface preparation in addressing this issue, whereas Pichette and coworkers suggest the use of silver and copper metals in preventing biofilm formation and algal growth [63]. The anti-fouling properties of various antibiotics (i.e., glutaraldehyde and chloramphenicol) have also been evaluated; however, these appear to be less effective in minimizing the growth of algae over longer periods of time [63]. Undoubtedly, the mechanism of biofilm formation is complex which seems to depend on a wide range of factors (i.e., type of microorganism, water composition, environmental conditions, material composition/structure, etc). Modifying the physical (i.e., surface structure and texture, etc) and chemical (i.e., doping with metal particles, etc) properties of existing MIR sensor materials is one method that can be used to reduce fouling and degradation processes. Likewise, the development of various micro-structured and superhydrophobic materials may offer some hope in tackling this difficult problem, however, it would be interesting to see how these and other materials perform analytically during the detection of hydrocarbon compounds in natural waters.

## 5. Conclusions

Effective environmental management requires reliable analytical tools that are capable of providing rapid and direct information on pollutant type and concentration. This review paper has shown that a polymer coated MIR-ATR sensor can be routinely used to screen for a vast range of environmentally significant compounds and contaminants. Compared to other technologies the MIR-ATR sensor offers much greater selectivity since the infrared spectrum is highly characteristic of a particular substance. Since the cavities of most of the polymeric materials are in the nanometre length range only small and hydrophobic compounds can be sampled. Although, there are many materials available for interacting with hydrocarbons, only a few can achieve ppb detection levels. Depending on the nature of the membrane the MIR sensor is able to simultaneously discriminate between many hydrocarbons from the same family due to their differing IR absorption spectra. However, one of the main challenges that have limited the long-term deployment of sensor devices in aquatic environments is the problem of biofouling. Various properties of a material (i.e., composition, structure and morphology) can be modified in order to reduce fouling problems and further research is needed to develop an MIR sensor which has improved long term stability in natural waters.

## Figures and Tables

**Table 1.** Analytical properties of a variety of infrared spectroscopic sensors for toluene in water.

**Method**	**IR Region**	**Detection Limit**	**Reference**
ATR	Middle	80 ppb	[20]
Fiber optic	Middle	1.3 ppm	[21]
Transmittance	Middle	6.9 ppm	[22]
Transmittance	Near	1.8 ppm	[23]

**Table 2.** The analytical performance of various polymers during ATR-MIR sensing of toluene in water.

**Polymer**	**Coating Method**	**Film Thickness**	**Detection Limit**	**Reference**
Ethylene-propylene copolymer	Drop cast followed by heating at 150°C	4.2 μm	80 ppb	[20]
Poly(acrylonitrile-co-butadiene)	Spin coated	5.1 μm	10 ppb	[29]
Teflon	Spin coated	5.1 μm	27 ppb	[16,29]
Polyisobutylene	Drop cast	NA	337 ppb	[30]

NA = not available

**Table 3.** The drinking water guideline value and solubility of some selected halogenated hydrocarbons.

**Halogenated hydrocarbon**	**Guideline value [46] (μg/L)**	**Solubility [47] (g/L)**
Carbon tetrachloride	4	0.65 (25°C)
1,2-Dibromo-3-chloropropane	1	1.23 (20°C)
1,2-Dibromoethane	4	3.1 (20°C)
1,2-Dichloroethane	30	8.6 (25°C)
1,2-Dichloroethene	50	6.4 (*cis*) (25°C) 4.5 (*trans*) (25°C)
Dichloromethane	20	17.6 (25°C)
1,2-Dichloropropane	40	2.74 (25°C)
1,3-Dichloropropene	20	2.7 (*cis*) (20°C) 2.8 (*trans*) (20°C)
Tetrachloroethene	40	0.21 (20°C)
Trichloroethene	20	0.128 (25°C)
Chloroform	300	8.0 (25°C)
Bromoform	100	3.0 (25°C)
